# Supplementary material for: Patterns of Wolf Dispersal Respond to Harvest Density across an Island Complex
Source: Animals (Basel). 2024 Feb 15;14(4):622. doi: 10.3390/ani14040622 (PMC10885989; doi:10.3390/ani14040622)
Supplement: Supplementary file 1 [file animals-14-00622-s001.zip › animals-2819712-supplementary.pdf]

Supplementary Table S1. Fall wolf population density estimates and predicted wolf population estimates using spatially-explicit capture-recapture (SECR), for Game Management Unit (GMU) 2 (9,025 km2), including Prince of Wales Island, Alaska, USA. Density values are presented ± SE (95% CI) and population estimates as 95% CI.

Year	Density (per 1,000 km^2)	Population estimate	95% CIs
2013	24.5 ± 6.8 (14.4–41.9)	221	130–378
2014	9.9 ± 3.0 (5.5–17.7)	89	50–159
2015	11.9 ± 2.7 (7.7–18.5)	108	69–167
2016	25.5 ± 3.1 (20.2–32.3)	231	192–285
2017	22.9 ± 2.6 (18.3–28.6)	225	198–264
2018	19.5 ± 2.1 (15.7–24.2)	187	147–236
2019	35.0 ± 4.1 (27.7–44.1)	316	250–398
2020	44.0 ± 4.8 (35.5–54.5)	386	321–472
2021	29.9 ± 3.3 (26.6–33.2)	268	216–332

Supplementary Table S2. Annual wolf harvest density by uniform coding unit (UCU) for Game Management Unit (GMU) 2 (9,025 km^2), including Prince of Wales Island, Alaska, USA during 2011–2021. Density was calculated as the number of wolves harvested in each UCU by year divided by UCU area.

UCU	2011	2012	2013	2014	2015	2016	2017	2018	2019	2020	2021
02ZX0901	0.0000	0.0134	0.0067	0.0000	0.0067	0.0134	0.0268	0.0201	0.0470	0.0201	0.0067
02ZX0902	0.0000	0.0143	0.0000	0.0000	0.0000	0.0214	0.0119	0.0167	0.0476	0.0119	0.0190
02ZX1003	0.0000	0.0058	0.0289	0.0347	0.0000	0.0000	0.0058	0.0000	0.0000	0.0116	0.0000
02ZX1105	0.0000	0.0000	0.0000	0.0168	0.0000	0.0077	0.0138	0.0031	0.0184	0.0046	0.0000
02ZX1106	0.0000	0.0000	0.0000	0.0254	0.0000	0.0085	0.0000	0.0000	0.0000	0.0000	0.0000
02ZX1107	0.0339	0.0100	0.0011	0.0056	0.0000	0.0011	0.0045	0.0033	0.0190	0.0156	0.0033
02ZX1108	0.0078	0.0000	0.0000	0.0000	0.0000	0.0000	0.0000	0.0000	0.0000	0.0027	0.0000
02ZX1209	0.0000	0.0000	0.0000	0.0000	0.0000	0.0000	0.0000	0.0000	0.0036	0.0000	0.0000
02ZX1210	0.0000	0.0000	0.0028	0.0000	0.0000	0.0000	0.0000	0.0000	0.0761	0.0000	0.0000
02ZX1211	0.0000	0.0000	0.0000	0.0000	0.0000	0.0000	0.0000	0.0124	0.0331	0.0372	0.0083
02ZX1212	0.0000	0.0000	0.0000	0.0000	0.0000	0.0000	0.0068	0.0000	0.0000	0.0000	0.0068
02ZX1213	0.0068	0.0000	0.0075	0.0075	0.0000	0.0000	0.0373	0.0000	0.0448	0.0075	0.0149
02ZX1214	0.0000	0.0000	0.0026	0.0000	0.0000	0.0000	0.0026	0.0103	0.0180	0.0000	0.0000
02ZX1315	0.0000	0.0026	0.0157	0.0000	0.0000	0.0104	0.0104	0.0052	0.0235	0.0183	0.0366
02ZX1316	0.0078	0.0000	0.0000	0.0000	0.0000	0.0000	0.0000	0.0060	0.0000	0.0000	0.0000
02ZX1317	0.0000	0.0069	0.0035	0.0000	0.0000	0.0000	0.0173	0.0035	0.0069	0.0069	0.0104
02ZX1318	0.0173	0.0218	0.0079	0.0000	0.0020	0.0060	0.0099	0.0060	0.0258	0.0079	0.0099
02ZX1319	0.0000	0.0046	0.0069	0.0000	0.0000	0.0000	0.0046	0.0023	0.0000	0.0000	0.0046
02ZX1323	0.0115	0.0069	0.0000	0.0000	0.0000	0.0000	0.0138	0.0000	0.0000	0.0000	0.0276
02ZX1332	0.0000	0.0000	0.0000	0.0114	0.0189	0.0000	0.0114	0.0076	0.0303	0.0000	0.0038
02ZX1420	0.0000	0.0317	0.0000	0.0000	0.0000	0.0159	0.0212	0.0265	0.0053	0.0317	0.0265
02ZX1421	0.0000	0.0107	0.0188	0.0000	0.0000	0.0054	0.0054	0.0027	0.0161	0.0027	0.0214
02ZX1422	0.0000	0.0040	0.0119	0.0020	0.0000	0.0000	0.0060	0.0119	0.0020	0.0000	0.0079
02ZX1524	0.0000	0.0000	0.0000	0.0000	0.0000	0.0000	0.0000	0.0000	0.0000	0.0000	0.0222
02ZX1525	0.0000	0.0158	0.0105	0.0000	0.0000	0.0000	0.0105	0.0000	0.0000	0.0000	0.0000
02ZX1526	0.0000	0.0000	0.0112	0.0000	0.0000	0.0000	0.0000	0.0000	0.0000	0.0112	0.0000
02ZX1527	0.0000	0.0057	0.0575	0.0000	0.0000	0.0000	0.0000	0.0000	0.0172	0.0172	0.0115
02ZX1528	0.0057	0.0000	0.0183	0.0000	0.0000	0.0000	0.0000	0.0000	0.0275	0.0000	0.0000
02ZX1529	0.0000	0.0000	0.0035	0.0000	0.0000	0.0000	0.0035	0.0000	0.0248	0.0106	0.0000
02ZX1530	0.0071	0.0041	0.0081	0.0000	0.0000	0.0000	0.0041	0.0000	0.0041	0.0000	0.0000
02ZX1531	0.0000	0.0000	0.0000	0.0000	0.0000	0.0000	0.0000	0.0155	0.0388	0.0078	0.0000
03ZX1906	0.0000	0.0000	0.0000	0.0000	0.0000	0.0000	0.0000	0.0000	0.0000	0.0000	0.0000
